# Instability of tear film and loss of meibomian glands in patients with *acne vulgaris*

**DOI:** 10.5935/0004-2749.2021-0038

**Published:** 2022-09-06

**Authors:** Yakup Acet, Leyla Bilik

**Affiliations:** 1 Department of Ophthalmology, Mardin Training and Research Hospital, Mardin, Turkey; 2 Department of Dermatology, Mardin Training and Research Hospital, Mardin, Turkey

**Keywords:** Acne vulgaris, Meibomian glands, Anterior chamber, Noninvasive break-up time test, Tear film, Acne vulgar, Glândulas de Meibomius, Câmara anterior, Teste não-invasivo de tempo de ruptura, Filme lacrimal

## Abstract

**Purpose:**

In this prospective study, we compared ocular clinical variables in patients with acne vulgaris with those of healthy controls. These variables included tear film break-up time, meibomian gland dropout rate, and anterior chamber parameters.

**Methods:**

Our sample comprised 73 eyes from 73 patients with acne vulgaris and 67 eyes from 67 healthy controls. All participants underwent a non-invasive first tear film break-up time test and the average tear film break-up time was evaluated. Meibography was used to identify any meibomian gland dropout. The parameters of the cornea and anterior chamber were measured using Scheimpflug topography imaging. Finally, the ocular surface disease index questionnaire was administered to score each participant on their subjective experience of ocular complaints.

**Results:**

The noninvasive first tear film break-up time values of the acne vulgaris Group and the control Group were 4.7 ± 2.8 and 6.4 ± 3.5 sec, respectively. There was a significant difference between the groups (p=0.016). The number of eyes with tear break-up at any time during the measurement period was also significantly higher in the acne Group (p=0.018). In the acne vulgaris Group, the mean meibomian gland dropout rates were 33.21 ± 15.5% in the upper lids and 45.4 ± 14.5% in the lower lids. In the control group, these rates were 15.7 ± 6.9% and 21 ± 9.7% respectively. Dropout was significantly higher in the acne group for both the upper and lower lids (p=0.000).

**Conclusion:**

We found impaired tear stability in patients with acne vulgaris and a high rate of meibomian gland dropout. These glands play a key role in tear stability and their dropout is likely to result in evaporative dry eye. Measurement of the variables in this study allows objective diagnosis of this condition using a non-invasive, dye-free methodology, with minimum contact.

## Abstract (pt)

**Objetivo:**

Neste estudo prospectivo, pacientes com acne vulgaris e indivíduos saudáveis do grupo controle foram comparados em relação ao tempo de ruptura do filme lacrimal, taxa de abandono de glândulas meibomianas e parâmetros da câmara anterior, usando o tempo de ruptura do filme lacrimal topográfico não invasivo, meibografia não invasiva e fotografia de Scheimpflug, respectivamente.

**Métodos:**

Setenta e três olhos de 73 pacientes com acne vulgaris e 67 olhos de 67 indivíduos saudáveis foram incluídos. Todos os participantes submetidos ao primeiro tempo de ruptura do filme lacrimal não-invasivo e ao tempo médio de ruptura do filme lacrimal não-invasivo foram avaliados pelo uso do tempo de ruptura do filme lacrimal; perda de glândulas meibomianas foram avaliadas por meibografia; os parâmetros da córnea e da câmara anterior foram medidos por fotografia de Scheimpflug; e, finalmente, as queixas oculares subjetivas foram pontuadas com o uso do questionário do Indice de doenças de superfície ocular.

**Resultados:**

Os valores do tempo de ruptura do primeiro filme lacrimal não-invasivo do Grupo com acne vulgaris e do Grupo controle foram 4,7 ± 2,8 e 6,4 ± 3,5 segundos, respectivamente, refererindo-se a uma diferença significativa entre os valores dos grupos (p=0,016). Qualitativamente, o número de olhos com ruptura lacrimal a qualquer momento durante o período de medição foi significativamente maior no grupo de pacientes. (p=0,018). No Grupo com acne vulgaris, a perda de glândulas meibomianas nas pálpebras superiores foi de 33,21 ± 15,5% e nas pálpebras inferiores foi de 45,4 ± 14,5%; por outro lado, no Grupo controle foi de 15,7 ± 6,9% e 21 ± 9,7% respectivamente; ambos os casos referem-se a uma diferença significativa entre os grupos (p=0,000).

**Conclusão:**

Encontramos estabilidade comprometida do filme lacrimal em pacientes com acne vulgaris. No entanto, o comprometimento foi de grau muito menor, em comparação com a taxa de perda das glândulas meibomianas que desempenham um papel fundamental na estabilidade do filme lacrimal. Esta condição pode ser documentada de forma objetiva - uma metodologia parcialmente sem contato, totalmente não-invasiva e livre de corantes.

## INTRODUCTION

Acne vulgaris (AV) is a leading cause of dermatology outpatient visits^(1)^. Its prevalence during adolescence has been reported, variously, as 18-85%^(2)^. Increased sebaceous gland secretion during adolescence due to changes in androgenic hormone release is believed to be the initial trigger in the complex inflammatory pathogenesis of acne formation^(3,4)^. Dysfunction of the pilosebaceous gland, influenced by hormones and bacteria, is also thought to play a role in genetically predisposed individuals^(5,6,7)^.

Meibomian glands (MG) are sebaceous glands in the eyelids. There are approximately 30 MG in the upper and 20 MG in the lower eyelid of a healthy eye^(5)^. The lipids produced by the MGs are essential to prevent rapid evaporation or instability of the eye’s protective tear film^(6)^. The meibomian glands are modified by the pilosebaceous glands responsible for the lipid secretions that prevent rapid tear evaporation^(7)^. Acne vulgaris is a lipid gland disorder and, since MGs are the sebaceous glands responsible for lipid production in tears, it should come as no surprise that MGs are affected by the factors involved in the pathophysiology of AV^(4,5,6,7,8,9)^. In addition, isotretinoin, which is used in the treatment of AV, has been shown by various studies to cause dry eyes^(7,8,9)^. To the best of our knowledge, no previous studies have compared noninvasive tear break-up time (T-BUT), meibomian gland function, and anterior chamber alterations in AV patients and healthy controls. This study aimed to compare corneal parameters, anterior chamber parameters, tear stability, and meibomian gland dropout, both objectively, using a corneal topography device, and subjectively, using ocular surface disease index (OSDI) scores of AV patients and healthy controls.

## METHODS

This study was carried out in accordance with the tenets of the 2013 version of the Declaration of Helsinki. Ethical approval was obtained from the research ethics committee of Harran University (#HRU/21.14.31). The required permissions were also obtained from Mardin’s Provincial Health Authority. Written informed consent for participation and publication was obtained from all participants. The study participants consisted of AV patients diagnosed and treated by a dermatologist at our institution (L.B.) (Group 1) and 67 healthy controls who attended our ophthalmology outpatient unit for routine eye check-ups (Group 2).

The exclusion criteria for Group 1 were the co- existence of systemic syndromes such as Sjögren’s syndrome or other cutaneous disorders such as contact dermatitis; a history of ocular or refractive surgery; the use of contact lenses; the use of eye drops for any reason; a previous diagnosis of dry eye; a refraction error of >1D spherical equivalent (sphere power + half of the cylinder power); and the presence of significant ptosis. All Group 1 participants had been newly diagnosed with AV. Individuals who did not receive medical treatment for AV were included in the study.

The exclusion criteria for Group 2 were a current or previous diagnosis of acne by a dermatologist; a current or previous systemic disorder such as Sjögren syndrome or cutaneous disorder such as contact dermatitis; a history of ocular or refractive surgery; the use of contact lenses; the use of eye drops for any reason; a previous diagnosis of dry eye; a refraction error of >1D in spherical equivalent (sphere power + half of the cylinder power); and the presence of significant ptosis.

In both groups, each patient underwent the following assessments and measurements in the order shown:

1. We began by assessing T-BUT using a corneal topography device (Sirius, Costruzione Strumenti Oftalmici [CSO], S.r.l, Italy) with a Scheimpflug camera function and an inbuilt keratoscope to eliminate the potential effect of blinking or eye rubbing during or after the slit-lamp or other clinic examination. Before the examination, each patient was asked to blink their eye twice upon instruction, after which they were to keep their eyes open and avoid blinking for as long as possible. Using the corneal topography device, T-BUT was assessed in the right eye of each participant.
2. Next, images were obtained using Scheimpflug tomography, and a keratoscope from the same device was used for corneal and anterior chamber analyses. The images were processed using specialized software that collected objective and numerical data. The ocular variables collected by the software were central corneal thickness (CCT), anterior chamber depth (ACD), anterior chamber volume (ACV), anterior chamber angle (ACA), and corneal volume (CV).
3. Meibography was then performed in the right upper and lower eyelid of the patient, with at least five images acquired for each eyelid. The three images with the best contrast and image quality were analyzed and marked manually by ophthalmologist (Y.A.). They were also processed using specialized software. The patient’s meibomian gland dropout rate was categorized using a five-point grading system as follows: grade 0, no dropout; grade 1, <25% dropout; grade 2, 26-50% dropout; grade 3, 51-75% dropout; grade 4, >75% dropout^(10)^. The average grade and dropout rate determined for the above-mentioned three images were recorded and later used for statistical analyses.
4. After these tests, the patient’s subjective experience of ocular symptoms was evaluated using the OSDI questionnaire. OSDI scores categorized the ocular surface as normal (0-12 points), mildly damaged (13-22 points), moderately damaged (23-32 points), or severely damaged (33-100 points).
5. Finally, a detailed ophthalmic examination was performed on participants who successfully performed the topographical measurements. Participants found at this stage to have blepharitis, conjunctivitis, concretion, or ocular surface irregularities due to corneal or eyelid deformities were excluded from the study.

Following these final exclusions, we were left with a total of 73 eyes from 73 patients in Group 1 and 67 eyes from 67 participants in Group 2.

The Sirius topography device (CSO) was used for meibography, T-BUT testing, and analysis of the cornea and anterior chamber. The device combines a Scheimpflug camera and a keratoscope. The Scheimpflug camera can perform a rapid 180° rotation, acquiring 25 cross-sectional images of the entire cornea and anterior chamber at 7-8° intervals, with real elevation points determined by assessment of 36,632 and 30,000 data points from the anterior and posterior corneal surfaces, respectively^(11,12)^.

**Figure 1:**
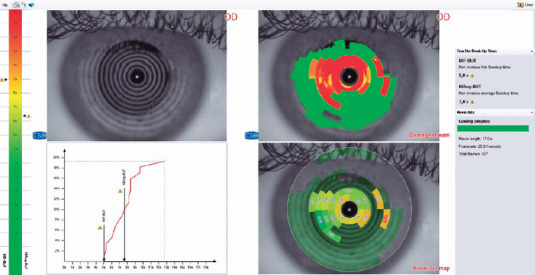
Printout of a T-BUT test and its general interpretation for the eye of a patient with acne vulgaris. The NIF-BUT in this patient was 5 sec, and the NIAvg-BUT was 7.6 sec. The initial tear film break-up occurred in the periphery of the inferonasal quadrant, with further peripheral tear film break-ups at 5.2 sec. However, the majority of the break-ups are shown in the image to have been located centrally and mid-peripherally. Tear film break-ups covered approximately 19% of the ocular surface analyzed. This information was obtained from the built-in software of the Sirius device used for ocular analysis. This device recorded and analyzed a total of 427 frames over 17 secs. T-BUT: Noninvasive tear break-up time. NIF-BUT: Non-invasive first break-up time NIAvg-BUT: Non-invasive average break-up time.

The video-keratoscope of the device acquires over 400 frames at a rate of 25 frames/sec for 17 sec. This yields the non-invasive first break-up time (NIF-BUT) and the non-invasive average break-up time (NIAvg-BUT) values in just one-tenth of a sec, after analyzing the data using specialized software. This device also uses imaging and quantitative data to determine the timing, quadrant, and degree of break-up on the ocular surface, as well as the location and time of all tear film break-ups, and the break-up intervals, using a color distribution chart (with the coloring of mild to severe break-ups graduating from green to red). If more than two tear break-up time measurements are performed, the Sirius automatically calculates the average NIF-BUT and NIAvg-BUT values^(13)^ (Figure 1).

**Figure 2:**
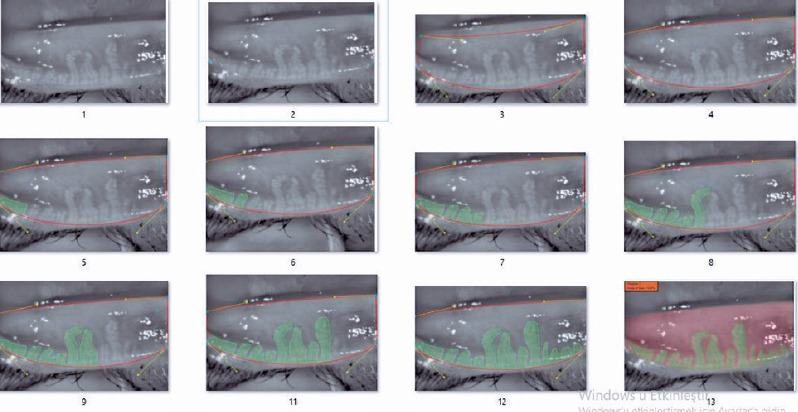
Lower eyelid meibography in an acne vulgaris patient with grade 3 dropout of 73% of the meibomian glands. This eyelid is used to illustrate the meibography process in the figure images. Image 1: Raw meibography images obtained under infrared light. Image 2: The corners are marked in accordance with eyelid anatomy. Image 3: A trapezoid roughly matching the eyelid anatomy is created by the device. Image 4: A trapezoid completely matching the eyelid anatomy is created by the device. Images 5–12: The location of the meibomian glands is identifed and automatically highlighted in green by the device. The remaining areas are highlighted in red and the rate of meibomian gland dropout is calculated by the software, both as a percentage and a grade. This procedure is repeated with three diferent images of the same eyelid and the average dropout rate for the three is recorded for statistical analyses.

The device renders the meibomian glands using an infrared (IR) light source. Then, four separate points are marked on the image to delineate the eyelid under examination. The area within these four points is depicted as a roughly trapezoid shape. The inner part of the trapezoid is manually screened for MG by the user of the device and automatically highlighted in green. The remaining part of the trapezoid, i.e., that which does not contain meibomian glands, is automatically highlighted in red. The proportion of the total trapezoid area that is colored red provides the MG dropout rate for the eyelid scanned, both as a percentage and as a grade on the five-point grading system described above^(10)^ (Figure 2).

The OSDI is a 12-item questionnaire designed for the rapid evaluation of a patient’s subjective experience of ocular symptoms consistent with dry eye disease. The OSDI aims to provide faster, easier, more reliable diagnoses of ocular surface diseases and to determine the presence of clinical symptoms of dry eye disease^(14)^.

### Statistical methods

The data in this study were described as the mean and standard deviation, median and range, and frequency and percentage. A Kolmogorov-Smirnov test was used to determine whether variables were normally distributed. Independent samples t-tests and Mann-Whitney U tests were used for between-group comparisons of quantitative data and chi-squared tests were used for between-group comparisons of qualitative data. Statistical analyses were performed using SPSS v. 27.0 (IBM Corp., Armonk, N.Y., USA) software.

## RESULTS

This prospective study included 73 patients diagnosed with AV (Group 1) who were age- and sex-matched with 67 healthy controls (Group 2). The mean age of group 1 was 24.5 ± 6.1 and that of group 2 was 25.7 ± 5.5 years. In Group 1, 76.7% of the participants were female compared to 83.6% in Group 2. Thus, the two groups were comparable in terms of age and gender.

### Tear film analysis

The average NIF-BUT of Group 1 was 4.7 ± 2.8 sec and that of Group 2 was 6.4 ± 3.5 sec. This was a statistically significant difference (p=0.016). The average NIAvg-BUT in Groups 1 and 2 were 7.9 ± 2.8 sec and 8.8 ± 3.1 sec, respectively. Although the NIAv-BUT was shorter in Group 1, the difference between groups was not significant (p=0.223). The proportion of eyes with at least one tear film break-up (referred to as “damaged” by the Sirius device) at any time during measurement was 79.4% in Group 1 and 61.2% in Group 2; this was a statistically significant difference (p=0.018). The ocular surface was divided into four 90° quadrants: the superonasal, inferonasal, inferotemporal, and superotemporal quadrants. The most common quadrants in which the first break-up occurred were determined but there was no significant difference between the two groups (p=0.501). However, in Group 1, 64.3% of the first tear film break-ups occurred in the inferior half (the inferotemporal and inferonasal quadrants combined) compared to 41.8% in Group 2. Inferior quadrant involvement was more common in both groups (Figure 1 and Table 1).

**Table 1:** Comparative analysis of clinical measures of ocular condition between patients with acne vulgaris and healthy controls.

		Patient group			Control group			p-value	
		Mean ± SD/ n-%		Median	Mean ± SD/ n-%		Median		
Damaged	(-)	15	20.6		26	38.8		**0.018**	X^2^
	(+)	58	79.4		41	61.2			
GUAD	SN	5	8.5		7	17.1		0.501	X^2^
	IN	22	37.3		15	36.6			
	IT	25	42.4		13	31.7			
	ST	7	11.9		6	14.6			
SM grade	I	27	37.0		62	92.5		**0.000**	X^2^
	II	34	46.6		5	7.5			
	III	12	16.4		0	0.0			
IM grade	I	7	9.6		48	71.6		**0.000**	X^2^
	II	37	50.7		18	26.9			
	III	29	39.7		1	1.5			
NIF-BUT		4.7 ± 2.8		4.0	6.4 ± 3.5		5.7	**0.016**	m
NIAvg -BUT		7.9 ± 2.8		7.4	8.8 ± 3.1		8.4	0.223	m
SM score		33.0 ± 1.,5		30.2	15.7 ± 6.9		14.2	**0.000**	m
IM score		45.4 ± 14.5		44.2	21.0 ± 9.7		19.4	**0.000**	m
CCT		538.1 ± 34.1		538.0	536.1 ± 31.8		535.0	0.723	t
ACD		3.12 ± 0.27		3.13	3.10 ± 0.29		3.16	0.801	m
ACV		168.0 ± 27.7		167.0	170.0 ± 26.0		176.0	0.666	t
ACA		44.0 ± 5.6		45.0	42.9 ± 6.0		44.0	0.230	m
CV		57.4 ± 3.3		57.5	57.1 ± 3.1		57.1	0.587	t
OSDI	Normal	47	64.4		51	76.1		0.103	X^2^
	Mild	12	16.4		11	16.4			
	Moderate	11	15.1		2	3.0			
	Severe	3	4.1		3	4.5			

t= t-test, m= Mann-Whitney u-test, X^2^= Chi-square test.

“Damaged” indicates the occurrence of breakup during measurement. It is a qualitative value.

GUAD= “Quadrant” refers to each 90°quadrant of the corneal surface, ACA= anterior chamber angle; ACD, anterior chamber depth; ACV= anterior chamber volume; CCT= central corneal thickness; CV= corneal volume; IM grade= inferior meibography grade; IM score= inferior meibography MG dropout percentage; MG= meibomian gland; NIAvg-BUT= non -invasive average break-up time; NIF-BUT= non-invasive first break-up time; OSDI= ocular surface disease index; SM grade= upper eyelid superior meibography grade; SM score= superior meibography MG dropout percentage.

OSDI questionnaire scores**:** normal (0–12 points), mild ocular damage (13-22 points), moderate ocular damage (23-32 points), severe ocular damage (33-100 points).

### Results of corneal and anterior chamber analyses

The average central corneal thicknesses (CCT) in Groups 1 and 2 were 538.1 ± 34.1 and 536.1 ± 31.8, respectively. These were not significantly different (p=0.723). Also, the ACD, ACV, ACA, and CV were comparable between the two study groups, with no significant between-group differences (p=0.801, p=0.666, p=0.230, and p=0.587, respectively) (Table 1).

### OSDI score analysis

Normal, mildly elevated, moderately elevated, and severely elevated OSDI scores were found in 47 (64.4%), 12 (16.4%), 11 (15.1%), and three (4.1%) of the patients in Group 1, respectively. The corresponding figures in Group 2 were 51 (76.1%), 11 (16.4%), two (3%), and three (4.5%), respectively. The OSDI scores were comparable between the two study groups, with no significant between-group differences (p=0.103) (Table 1).

### Meibography analyses

Based on the five-point grading system^(10)^, the superior meibography (SM), which is that of the upper eyelid, showed grade 1, grade 2, and grade 3 dropouts in 27 (37%), 34 (46.6%), and 12 (16.4%) patients, respectively. In Group 2, these grades occurred in 62 (92.5%), 5 (7.5%), and 0 (0.0%) of participants, respectively. The difference between groups was statistically significant (p=0.000). The inferior meibography (IM), which is that of the lower eyelid, showed grade 1, grade 2, and grade 3 dropouts in seven (9.6%), 37 (50.7%), and 29 (39.7%) of the patients in Group 1, respectively. In Group 2, these grades occurred in 48 (71.6%), 18 (26.9%), and one (1.5%) of the participants, respectively. Again, there was a significant difference between the two groups (p=0.000) (Table 1).

**Figure 3:**
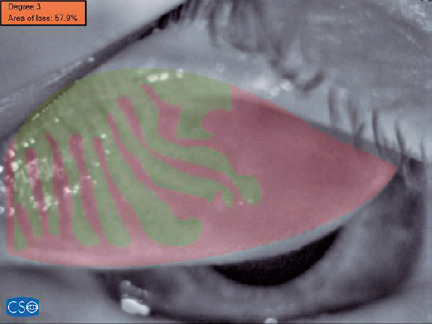
Superior (upper eyelid) meibography image from a patient with acne vulgaris. This eyelid was classifed with a grade 3 rate of meibomian gland dropout (51–75% dropout). The specific percentage of meibomian glands lost from this eyelid was 57.9%.

The mean MG dropout percentages found by superior meibography (SM) were 33.0 ± 15.5% in Group 1 and 15.7 ± 6.9% in Group 2 (p=0.000). The average percentages found by inferior meibography were 45.4 ± 14.5% in Group 1 and 21 ± 9.7% in Group 2 (p=0.000). The difference between groups was statistically significant for both eyelids (Table 1, Figures 2 and 3).

## DISCUSSION

In this study, we found lower NIF-BUT values in AV patients than in healthy controls, indicating tear instability in this patient population. A study by Özdemir et al. found abnormal tear film BUT in approximately 21% of patients diagnosed with nodulocystic acne^(15)^. In the current study, 79% of our AV patients were found to have at least one tear film break-up during the test period. While our results found an abnormal BUT of <17 sec, previously reported figures for this demographic have been <10 sec^(15)^. A short NIF-BUT is indicative of dry eye. Acne vulgaris is an inflammatory disorder of the pilosebaceous glands. It predominantly occurs on the face, although other areas may also be affected^(5)^. AV is the result of a complex interaction between androgens and the *Propionibacterium acnes* bacteria in those genetically predisposed to the condition. It occurs at the cutaneous level of the pilosebaceous glands, which can be found close to the surface of all human skin except for the palms, including the eyelids^(5,6,7)^. The pilosebaceous glands of the eyelids are modified and this slightly altered form are known as meibomian glands. These are responsible for the lipid secretions that prevent tears from evaporating too rapidly to keep the eyes moist^(5)^. Since these secretions of the meibomian gland are the primary factor in tear film stability, its impairment is known to occur in dry eye disease. As our results show, tear film stability is diminished in many patients with AV, therefore; it is plausible to assume that meibomian gland secretion may also be diminished in those with acne.

Until now, most studies of correlations between AV and eye conditions have focused on the ocular side effects of isotretinoin, a treatment for AV that has been found to induce, or at least, significantly contribute to, dry eye disease in AV patients treated with it ^(7,8,9)^.

OSDI scores, reflecting the subjective experience of ocular clinical symptoms, did not differ significantly between our healthy controls and AV patients, of whom, 65% had normal OSDI scores. Özdemir et al. had very similar results, with normal OSDI scores in 67% of their participants^(15)^. The mean difference in NIF-BUT between our two groups was 1.7 sec and, although this difference was statistically significant, it was much less pronounced when the extent of MG dropout was taken into consideration. A multitude of factors contribute to the structure, stability, and equilibrium of the tear film, including androgenic hormones^(16)^, immune responses^(17)^, tear proteins^(17)^, microbes, and blinking^(18)^. These elements also collectively determine the liquid and lipid components of the tear film^(19,20)^. Previous research into MG loss has found a decline in MG with age ^(21)^. Other factors found to be associated with MG loss include contact lens use^(22,23)^, allergies^(24)^, glaucoma medications and surgery^(25,26)^, and rosacea^(27)^. Similarly, we observed a significant loss in MG in patients with AV. It is possible that severe MG loss, such as that observed in our AV patients, could be compensated for by the liquid component of the tear film, and this may explain the lack of significant symptoms (represented by OSDI scores) in our patients. The advanced MG loss seen in our newly diagnosed AV patients indicates that pathologies related to the ocular surface must begin very soon after AV onset. However, we postulate that compensatory mechanisms may mask these ocular issues. The use of medication such as isotretinoin exacerbates the pathology sufficiently to override these compensatory mechanisms and trigger dry eye symptoms and tear film disruption.

Our AV and control groups were also comparable with regard to corneal and anterior chamber measurements. The average CCT was 538 in the current study, which is in accord with the results of previous studies^(28)^.

A limitation of our study relates to the fact that no tests for determining the effect of compensatory mechanisms have been performed.

Although a degree of tear film impairment was found to occur in patients with acne vulgaris, this is relatively mild given the degree of MG loss. We believe that AV patients should be closely monitored for MG loss and the resulting dry eye disease. This study has also shown that a corneal topography device represents a non-invasive and dye-free means of diagnosing MG loss and analyzing the tear film layer.
